# The Massachusetts Emergency Medical Service Stroke Quality Improvement Collaborative, 2009–2012

**DOI:** 10.5888/pcd10.130126

**Published:** 2013-09-26

**Authors:** Denise H. Daudelin, Erin R. Kulick, Katrina D’Amore, Jennifer S. Lutz, Mirian T. Barrientos, Kathy Foell

**Affiliations:** Author Affiliations: Denise H. Daudelin, Jennifer S. Lutz, Tufts Medical Center, Boston, Massachusetts; Erin R. Kulick, Katrina D’Amore, Mirian T. Barrientos, Kathy Foell, Massachusetts Department of Public Health, Boston, Massachusetts.

## Abstract

**Introduction:**

Quality improvement collaboratives are a popular model used to address gaps between evidence-based practice and patient care. Little is known about use of such collaboratives in emergency medical services, particularly for improving prehospital stroke care. To determine the feasibility of using this approach to improve prehospital stroke care, we conducted a pilot study of the Emergency Medical Services Stroke Quality Improvement Collaborative.

**Methods:**

Seventeen Massachusetts emergency medical service agencies participated in the quality improvement collaborative pilot project. We identified 5 prehospital stroke performance measures to assess the quality of prehospital care, guide collaborative activities, and monitor change in performance over time. During learning sessions, participants were trained in quality improvement and performance measurement, analyzed performance measure results, and shared successes and challenges. Focus groups were conducted to understand participants’ experiences with the collaborative.

**Results:**

Participating emergency medical service agencies collected stroke performance measures on 3,009 stroke patients during the pilot study. Adherence to 4 of 5 performance measures increased significantly over time. Participants acknowledged that the collaborative provided them with an efficient and effective framework for stroke quality improvement and peer-learning opportunities.

**Conclusion:**

As evidenced in Massachusetts, quality improvement collaboratives can be an effective tool to improve prehospital stroke care. The data collected, improvements made, participation of emergency medical service agencies, and positive experiences within the collaborative support the continued use of this approach.

## Introduction

Stroke is among the leading causes of severe long-term disability and death in the United States, ranking fourth as a cause for mortality behind heart disease, cancer, and chronic lower respiratory disease (1). An estimated 795,000 people suffer from a new or recurrent stroke each year; stroke mortality was approximately 134,000 in 2008 (1). In Massachusetts, stroke continues to be the third leading cause of death and disability, contributing to over 17,000 hospitalizations and 2,500 deaths annually (2).

Emergency medical services (EMS) are the first point of contact for approximately half of all patients with acute stroke symptoms in Massachusetts (3). Patients transported by EMS have faster prehospital transport, in-hospital evaluation, and treatment times (4–6). Patients arriving with suspected stroke also comprise most cases presenting in the 3-hour window for intravenous tissue plasminogen activator (IV t-PA), the only Food and Drug Administration–approved treatment of acute ischemic stroke (3,7).

Guidelines that focus on the importance of stroke recognition, rapid triage and transport, and hospital prenotification exist for EMS assessment and management of patients with suspected stroke (8,9); however, there are no nationally recognized prehospital stroke performance measures for gauging guideline adherence. Research suggests that prehospital stroke care is inconsistent (10,11), and gaps in stroke care significantly affect timely treatment on arrival at a hospital (12). However, EMS agencies often have few resources available to identify and address gaps in performance.

Quality improvement collaboratives (QICs) are a popular method in use in health care to address the gaps between evidence-based practice and clinical patient care. QICs bring together providers from different organizations to work in a structured way to improve the quality of their patient care. Through a series of meetings, participants learn about evidence-based best practices and quality improvement (QI) methods while sharing their own experiences and making changes in their organizations. The Massachusetts EMS Stroke QIC was based on the Institute for Healthcare Improvement’s (IHI) Breakthrough Series approach, which is designed to help organizations make “breakthrough” improvements by creating a forum in which participants can simultaneously learn improvement strategies from each other and from experts (13). Hospitals, clinics, primary care practices, and nursing homes frequently use this process. Evidence of the impact of QICs in these settings is positive but limited (14). Little is known about the use of this approach in EMS.

In 2008, the Massachusetts Department of Public Health’s (MDPH’s) Heart Disease and Stroke Prevention and Control Program began a stroke QIC pilot, modeled after their Paul Coverdell National Acute Stroke Registry QIC, the Stroke Collaborative Reaching for Excellence (SCORE) (15). Its purpose was to understand how the collaborative model could be used to address gaps in prehospital stroke care. The goal of this pilot was 2-fold: to determine the feasibility of an EMS collaborative and to assist EMS providers with implementing changes that would lead to consistent delivery of high-quality stroke care. This article describes the formation and characteristics of the QIC, the development and use of stroke performance measures, and best practices and challenges in using the collaborative method for EMS clinical QI.

## Methods

The EMS Stroke QIC began by developing a set of prehospital stroke performance measures. Participants were recruited and in-person meetings were conducted to educate participants in QI and performance measurement and to share successes and challenges among members. Lastly, performance measure results were collected and analyzed to determine the success of the pilot. The QIC leadership team included representatives from MDPH, statewide leaders in stroke care, and a health services researcher with QI and clinical experience. MDPH determined that institutional review board approval was not required for this project.

### Performance measures

A set of process-based stroke performance measures was outlined to measure the quality of prehospital care, guide the activities of the QIC, and monitor change in performance over time. Performance measures and intervention strategies were developed based on guidelines supported by the American Heart Association/American Stroke Association (9,16) and the National Association of EMS Physicians (17). These evidence-based and best-practice interventions focused on correctly assessing a patient’s condition, providing appropriate prehospital care, and promoting timely IV t-PA therapy for ischemic stroke for patients upon arrival at the emergency department (ED) (18,19).

A 15-member expert panel, comprising EMS and in-hospital stroke care stakeholders (neurologists, EMS leaders, state regulators, and ED physicians) reviewed relevant literature and guidelines around acute stroke care and developed a list of 10 candidate measures. Each member rated the measures based on 6 dimensions of quality: importance, contribution to better patient outcomes, opportunity for improvement, clarity and completeness, actionability, and overall usefulness. Five measures were selected for use in the pilot project: 1) stroke screening performed, 2) blood glucose tested, 3) time last-known-well documented, 4) time of symptom discovery documented, and 5) stroke prenotification to hospital (Table 1). Stroke screening and blood glucose testing were selected to ensure appropriate identification of stroke patients and to rule out stroke mimics. The Cincinnati Stroke Scale was used for the measure because it is required by the Massachusetts EMS Stroke Point of Entry Plan (10,20,21). Assessment and documentation of time last-known-well and time of symptom discovery were chosen to help determine patient eligibility for IV t-PA, and stroke prenotification was chosen to promote rapid evaluation and treatment on arrival at the ED. Patient inclusion criteria included cases with primary or secondary clinical impression of stroke, suspected stroke, or transient ischemic attack (TIA). The expert panel determined TIA to be an inclusion criterion and treated them as stroke because it is not always clear whether symptoms will resolve. All selected measures fell within the scope of care provided by Massachusetts Advanced Life Support and Basic Life Support Emergency Medical Technicians (EMTs) and the Massachusetts EMS Stroke Point of Entry plan (21).

**Table 1:** Stroke Performance Measures and Measure Definitions from the Masschusetts EMS Stroke Quality Improvement Project, 2009–2012

Measure Name	Measure Definition
**Stroke screening performed**	Percentage of patients with a clinical impression of stroke, possible stroke, or TIA who have a Cincinnati stroke screen documented.
**Blood glucose tested**	Percentage of patients with a clinical impression of stroke, possible stroke, or TIA who have blood glucose level documented by capillary blood glucose measurement.
**Time-last-known-well documented**	Percentage of patients with a clinical impression of stroke, possible stroke, or TIA who have a documented time at which the patient was last known to be without the signs and symptoms of the current stroke or at his or her prior baseline.
**Time of symptom discovery documented**	Percentage of patients with a clinical impression of stroke, possible stroke, or TIA who have a time of symptom discovery documented.
**Stroke prenotification to hospital**	Percentage of patients with a clinical impression of stroke, possible stroke, or TIA for whom stroke specific prenotification was called to the receiving emergency department and documented.

Abbreviation: EMS, emergency medical services; TIA, transient ischemic attack.

### Recruitment

EMS agencies were recruited to participate in the QIC from July through December 2009; data collection began upon recruitment. A purposeful sample was selected to include a variety of organization types, sizes, and geographic locations across the state. Agencies were required to have a well-established electronic patient care record (ePCR) system capable of collecting performance measure data elements as well as an active QI program with dedicated staff resources. Each agency designated a QI team to attend collaborative meetings and participate in improvement activities. Participation was voluntary and agencies were not compensated for their participation in the QIC. At the time of enrollment, agencies completed a baseline survey that inventoried agency characteristics, existing QI infrastructure, and policies around stroke care.

### Data collection

An automated QI information system (Clinical Care Systems Incorporated, Boston, Massachusetts) served as the data repository and automatically managed performance measure calculations. Agencies adapted their existing ePCR systems to electronically transmit all patient records to the central Web-based QI data repository. The repository identified patients with a primary or secondary clinical impression of stroke, suspected stroke, or TIA and automatically compared documented patient care against performance-measure criteria. Patient records identified as not meeting 1 or more performance measure criteria were flagged for manual review by the agency to determine if the record’s narrative description contained the missing data or to verify that the case did not meet performance criteria.

Monthly feedback reports based on measure adherence were available to agencies through the automated QI system. Participants had access to their own aggregate and patient level results through a password-protected, secure website. The website displayed the results of each of the 5 measures over time by using easy-to-interpret line and bar charts. These feedback reports offered the opportunity for QI staff within the EMS agency to follow up with EMTs about the care provided.

### Collaborative improvement activities

Structured activities were conducted to promote collaborative learning, support, and exchange of ideas and insights across EMS agencies. These included in-person learning sessions, individual calls, site visits, and training modules. At the time of recruitment, a site visit to the EMS agency explained the aims of the program and the expectations of the agency. After the agency started submitting data, a second site visit focused on data quality and validation. If requested, on-site continuing education events were hosted for agency staff and neighboring EMS agencies.

Learning sessions were held quarterly during which clinical content and QI experts provided both didactic sessions and hands-on exercises. Educational topics included evidence-based improvement strategies, methods for organizational change, and application of strategies in local settings. In addition, participants provided updates on their improvement activities to the larger group, shared their best practices, and received input from the collaborative on their challenges. Continuing education credits were offered as incentives for attendance.

Between learning sessions, each service received individual site calls. These coaching opportunities offered QI strategies specifically tailored to each EMS agency and its improvement focus at that time. The Plan-Do-Study-Act (PDSA) improvement model was introduced and small tests of change were used extensively (22).

### Performance measure analysis

Agency data were aggregated and analyzed quarterly from baseline (July through December 2009) to June 2012. In this time frame, 3,009 patients with stroke, suspected stroke, or TIA were transported by participating agencies. Agencies joined at different times and with different levels of submission during an initial 6-month period. For this reason, we chose to consider the first 6 months as baseline in order to give a clear indication of where all agencies were at the start. Joinpoint regression analyses (Joinpoint Regression Program, Version 3.43; Statistical Methodology and Applications Branch, Surveillance Research Program, National Cancer Institute, Washington, DC) were used to test for significant trends and to determine the average percentage change over time. The overall trends for measure adherence by quarter were computed, and significance was set at *P* < .05.

### Participants’ perception of the collaborative

To understand participants’ experiences and perceptions of the QIC, 2 focus groups were conducted by an external evaluation firm in May 2011. Focus groups were held either preceding or following a quarterly learning session both for ease of recruitment and to limit burden on participants. The interview guide consisted of 11 questions covering 5 main topics: 1) reasons for participation in the QIC, 2) successes and challenges, 3) experiences and level of satisfaction, 4) use of data and information, and 5) effect on relationships within their community (Appendix). Two leaders facilitated the focus groups; 1 conducted the groups and the other took detailed notes of participant responses. The evaluators used a systematic and rigorous process to achieve the qualitative analysis of data from the 2 groups. Notes taken were reviewed and revised by both leaders as needed, then transferred into the qualitative data analysis tool Atlas.ti (Version 4.2, Scientific Software Development, Berlin, Germany). Question coding was constructed to identity themes for each objective, and quotes were selected that illustrated those themes. The evaluation team used a consensus approach to identify the most important themes and quotes.

## Results

Seventeen EMS agencies participated in the pilot phase of the Massachusetts EMS Stroke QIC from July 2009 through June 2012. In a baseline survey, (n=14 respondents), organizations classified themselves as private, municipal, fire department–based, or volunteer EMS services from rural, urban, city, or suburban settings throughout Massachusetts (Table 2). QI teams ranged from 1 to 4 members and included EMS QI coordinators, managers, medical directors, and hospital-based EMS and stroke coordinators. Thirteen of the agencies were considered active participants (ie, attended at least 50% of learning sessions, tracked measure performance, and regularly engaged in QI activities).

**Table 2:** Characteristics of Agencies Participating in the Massachusetts EMS Stroke Quality Improvement Collaborative^a^, 2009-2012

Agency Characteristic^b^	Median (range per agency)	N, overall
**Ambulance level of service (number of vehicles per agency)**		
Basic life support	1.5 (0–20)	50
Advanced life support	2.5 (0–15)	56
**EMT level of service (number of EMTs per agency)**		
EMT, basic	21 (0–400)	753
EMT, intermediate	0 (0–18)	54
EMT, paramedic	18 (3–90)	357
**Annual emergency call volume (number of dispatches per agency)**	3,377 (400–100,000)	170,939

Abbreviation: EMS, emergency medical services; EMT, emergency medical technician.

a Data based on submitted baseline surveys from 14 participating emergency medical services agencies.

b EMS agencies classified themselves as fire department–based (n = 10), municipal (n = 6), and private (n = 3); by geographic location if they were rural (n = 6), suburban (n = 5), city (n = 4), and urban (n = 3). Agencies could classify themselves into more than one category.

Performance measures were collected and analyzed for 3,009 patients (agency range, 2–1,560) with a clinical impression of stroke, suspected stroke, or TIA. Four of the 5 stroke quality performance measures (blood glucose tested, time last-known-well, documented, time of symptom discovery documented, and stroke prenotification to hospital) showed significant trends of improvement over time (Figure); all 5 performance measures showed an increase in adherence over time (Table 3).

**Figure:**
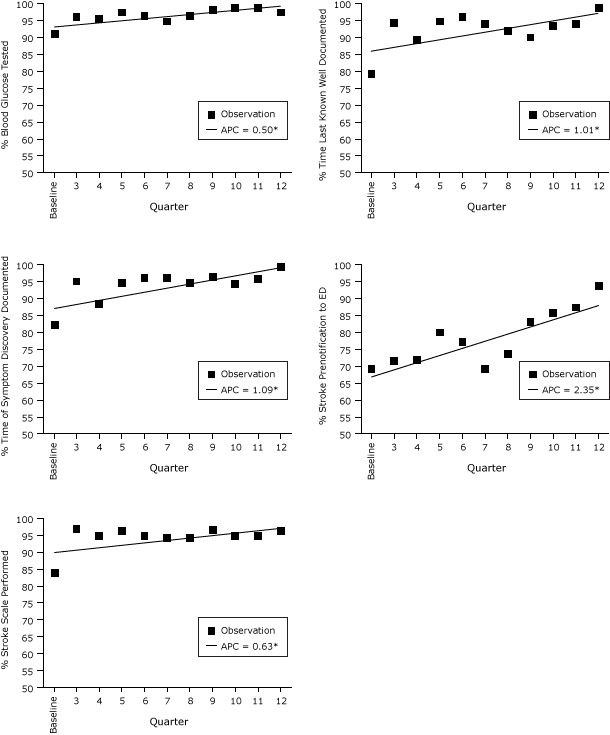
Performance measures adherence and average percentage change over time, Massachusetts Emergency Medical Service Stroke Quality Improvement project, July 2009–June 2012. Asterisks indicate measures with significant changes from baseline. Abbreviation: APC, average percentage change. **Table:** 

Action	Quarter											Average Percent Change in Range of Modeled Values, Baseline to Quarter 12

	Baseline (Q1-Q2)	3	4	5	6	7	8	9	10	11	12	
Blood glucose tested	91.2	96.2	95.5	97.3	96.5	94.6	96.5	98.2	98.8	98.8	97.3	93.2–98.9
Time-last- well-known documented	79.4	94.5	89.5	94.6	94.1	93.9	91.9	90.1	93.3	93.9	98.6	86.1–97.1
Time of symptom discovery documented	82.4	95.1	88.4	94.6	96.1	96.2	94.6	96.6	94.5	95.8	99.4	87.2–99.3
Stroke prenotification to hospital	69.1	71.7	71.8	79.9	77.1	69.3	73.6	83.0	85.6	87.3	93.6	66.6–88.0
Stroke screening performed	93.8	97.2	95.1	96.2	94.9	94.2	94.1	96.7	94.8	95.1	96.3	90.4–97.5

**Table 3:** Massachusetts Emergency Medical Services (EMS) Stroke Quality Improvement Collaborative Performance Measure Adherence Trends, Masschusetts EMS Service Stroke Quality Improvement Project, 2009–2012

Performance Measure	Baseline Measure Adherence, %	Quarter 12 Measure Adherence, %	Average Quarterly % Change (95% CI)	*P* Value^a^
Blood glucose tested	91.2	97.3	0.5 (0.2–0.8)	.01^b^
Time-last-known-well documented	79.4	98.6	1.01 (0.2–1.9)	.04^b^
Time of symptom discovery documented	82.4	99.4	1.1 (0.4–1.8)	.02^b^
Stroke prenotification to hospital	68.5	93.6.	2.4(1.5–3.8)	.001^b^
Stroke screening performed	83.8	96.3	0.6 (−0.2 to 1.3)	.16

Abbreviation: EMS, emergency medical services.

a *P* values were calculated by using joinpoint regression analyses.

b Significant at *P* < .05.

Eleven EMTs representing 11 different agencies and 1 hospital EMS coordinator attended the focus groups. After examination of the data, results were consolidated into 2 emerging themes: incentives for participation in the collaborative and barriers to participation in the collaborative.

The overarching theme of the focus groups was that the QIC provided participants with an efficient and effective framework for stroke QI and the opportunity to share and learn from each other. Benefits of continued participation in the QIC included the introduction of new stroke care practices, opportunities for statewide networking, and improved working relationships with hospital staff and medical directors. Barriers to participation were centered on the availability of resources, specifically staff time.

Participants described benefits of and barriers to QIC participation:

### Benefits of participation

- Provided a framework for stroke data collection and analysis using performance measures.
- Provided data to illustrate improvements in patient care. “Hospitals collect data on everything but no one was asking for prehospital data. The data we are showing makes a difference in the patient ahead of time.”
- Offered opportunity for learning and sharing best practices. “I like that nothing is proprietary. Presenters will email their presentation to us and allow us to come back and use it in our own department.”
- Enabled sharing of information, best practices, and challenges among various EMS agencies that would not normally collaborate.
- Provided networking opportunities between EMS personnel, clinicians, state agency staff, and physicians. “There is a great cross-section of people both geographically and from a variety of services and expertise.”
- Allowed participants to become familiar with QI tools and use of new strategies to successfully implement change.

### Barriers to participation

- Competing project priorities and other quality improvement responsibilities.
- Limited staff time and resources for stroke care. “We have other projects going on, like STEMI [ST Segment Elevation Myocardial Infarction], and our time has to be split. The [EMS] department does not have a lot of resources. However, the technology and automation helps.”
- Difficulty obtaining buy-in from leadership at the start of the project.
- Logistical challenges to travel, lack of staff coverage, and funding for the additional cost of overtime, which limited attendance at in-person meetings.

## Discussion

QICs are a widely accepted approach in many areas of health care but have not been used previously in the prehospital setting. Furthermore, numerous efforts are ongoing nationally to improve stroke care. Most prehospital QI efforts address stroke identification in the field through a series of educational strategies and the subsequent implementation of stroke screening tools (23–27). A successful model in North Carolina has presented EMS agencies with tool kits containing stroke performance measure reports for benchmarking and suggestions for improvement (28,29).

Our pilot EMS Stroke QIC was conducted with 17 agencies throughout Massachusetts. In the acute hospital setting, programs such as the Stroke Collaborative Reaching for Excellence (SCORE), the model for this pilot study, have successfully improved the in-hospital care of stroke patients by using a QIC (3,15). Participating EMS agencies attended learning sessions, engaged in collaborative improvement activities, used rapid-cycle improvement techniques, and evaluated the clinical care provided to stroke patients by using 5 standardized performance measures (Table 1).

The 6 components of a successful QI model are 1) clear choice of improvement subject, 2) participants’ objectives and capacity to participate, 3) clear roles and expectations, 4) activities to foster learning, 5) measurable and achievable improvement targets, and 6) the ability of participants to collect and use data and make changes in care (30). Implementing the QI model in the EMS setting was challenging because improvement strategies differed across agencies with organizational culture, environment, and budget. For example, some participants revised internal stroke policies, and others focused on implementing change through regional policies. Some used a more patient-centered QI approach and audited recorded Central Medical Emergency Direction (C-MED) radio transmissions to monitor the clarity and completeness of stroke prenotification calls to hospitals. In 1 location, a policy of direct transport from EMS to computed tomographic scan was implemented thanks to the creation of an EMS–hospital ED partnership focused on expediting patient care. Consistent in each agency, however, was the use of QI training, performance measure feedback, one-on-one QI coaching, and the use of patient-specific follow-up. Barriers to implementation of these QI strategies were identified at each step by participants and tested through PDSA cycles, and results and best practices were shared within the collaborative. The practice of sharing at learning sessions often led to the adoption of activities by other agencies. A successful stroke alert policy, executed and evaluated in 1 state EMS region, has since been implemented in 2 other regions.

Four of the 5 performance measures used to gauge the quality of prehospital care and guide the activities of the QIC participants showed significant and sustained improvement during the project (Figure). The fifth measure, stroke screening, also increased over time although not significantly. This may be due to the high rate of adherence at the start of the project. The focus groups indicated that the major barriers to participation included lack of resources, competing QI priorities, and staffing conflicts. As the project proceeded, attempts were made to minimize resources needed to participate, and researchers made individual site calls more frequently than conducting on-site meetings. In-person learning sessions continued to provide opportunities for participants to learn from their peers and from experts in the field of stroke care, an experience not previously available to most EMS agencies. This pilot project illustrates that despite limitations, participants overall can learn and apply new QI methods, while sustaining improvement. Participants recruited at the start of the pilot continued their voluntary participation throughout. Our results suggest that the QIC approach is feasible in the prehospital setting and may be an effective and sustainable model for EMS QI.

This study had several limitations. Performance-measure data were compiled by using data extracted from ePCR systems; therefore, the data quality and accuracy could not be fully assessed. Monitoring and improving data quality was an ongoing effort throughout the project, including using required data fields and reading unstructured narrative text when performance measures were not met. Furthermore, 2 of the performance measures, time last-known-well and time of symptom discovery, were not collected for all patients at the start of the study. Barriers to collecting these elements were identified as 1) data mapping issues within agency ePCR systems, 2) unclear or incomplete documentation, and 3) use of unsearchable narrative text fields to enter the measures. Strategies to address these barriers included 1) bundling stroke care data elements in a single location to make data submission easier and more complete, 2) requiring completion of stroke care data elements before closing the electronic record, and 3) frequent feedback to EMTs on data quality.

Factors other than the QI activities described may have influenced our results. Selected EMS agencies were favored, in part, because of their ePCR and information technology resources. Although many types and sizes of agencies were represented, agencies without an ePCR or automated QI system may have different results. Unlike other QICs, such as IHI’s Collaborative model, which lasts 12 to 15 months, the results reported here occurred over 36, months giving participants ample opportunity to implement changes. Four of the 5 measures brought about improvement within the first 12 months, suggesting that shorter QICs may still be effective.

Despite these limitations, we believe that using a collaborative approach to QI in the EMS setting is an effective tool to improve prehospital stroke care. The scope of the data collected, the significant improvements made in performance measure adherence, the sustained participation of EMS agencies, and the positive experiences of participants in the collaborative encourage us to continue to use this approach in EMS. Future steps will include the development of the project beyond the pilot stage with the recruitment of a second cohort of EMS agencies and continued work with the pilot cohort to implement QI strategies. The EMS QIC will also begin to work the SCORE in-hospital collaborative to improve care transitions from EMS to ED and ultimately develop a cohesive stroke system of care in Massachusetts.

## Appendix. Focus group question guide, Emergency Medical Services (EMS) Stroke Quality Improvement Collaborative.

This file is available for download as a Microsoft Word document

## References

[1] Roger VL , Go AS , Lloyd-Jones DM , Benjamin EJ , Berry JD , Borden WB , Heart disease and stroke statistics — 2012 update: a report from the American Heart Association. Circulation 2012;125(1):e2–220. 10.1161/CIR.0b013e31823ac046 22179539 PMC4440543

[2] Massachusetts Department of Health Division of Research and Epidemiology, Bureau of Health Information, Statistics, Research and Evaluation. A decade of mortality in Massachusetts: 2000–2009. Boston (MA): Massachusetts Department of Health; 2012.

[3] Massachusetts Department of Public Health. Massachusetts Paul Coverdell National Acute Stroke Data; 2012. http://www.scorema.org. Accessed March 12, 2013.

[4] Ekundayo OJ , Saver JL , Fonarow GC , Schwamm LH , Zian Y , Zhao Z , Patterns of emergency medical services use and its association with timely stroke treatment: findings from Get With the Guidelines-Stroke. Circ Cardiovasc Qual Outcomes 2013;6(3):262–9. 10.1161/CIRCOUTCOMES.113.000089 23633218

[5] Evenson KR , Foraker RE , Morris DL , Rosamond WD . A comprehensive review of prehospital and in-hospital delay times in acute stroke care. Int J Stroke 2009;4(3):187–99. 10.1111/j.1747-4949.2009.00276.x 19659821 PMC2825147

[6] Mosley I , Nicol M , Donnan G , Patrick I , Kerr F , Dewey H . The impact of ambulance practice on acute stroke care. Stroke 2007;38(10):2765–70. 10.1161/STROKEAHA.107.483446 17717317

[7] Tissue plasminogen activator for acute ischemic stroke. The National Institute of Neurological Disorders and Stroke rt-PA Stroke Study Group. N Engl J Med 1995;333(24):1581–7. 10.1056/NEJM199512143332401 7477192

[8] Jauch EC , Saver JL , Adams HP , Bruno A , Connors JJB , Demaerschalk BM , Guidelines for the early management of patients with acute ischemic stroke: a guideline for healthcare professionals from the American Heart Association/American Stroke Association. Stroke 2013;44(3):870–947. 10.1161/STR.0b013e318284056a 23370205

[9] Schwamm LH , Pancioli A , Acker JE , Goldstein LB , Zorowitz RD , Shephard TJ , Recommendations for the establishment of stroke systems of care: recommendations from the American Stroke Association’s Task Force on the Development of Stroke Systems. Stroke 2005;36(3):690–703. 10.1161/01.STR.0000158165.42884.4F 15689577

[10] Kothari RU , Pancioli A , Liu T , Brott T , Broderick J . Cincinnati Prehospital Stroke Scale: reproducibility and validity. Ann Emerg Med 1999;33(4):373–8. 10.1016/S0196-0644(99)70299-4 10092713

[11] Kidwell CS , Starkman S , Eckstein M , Weems K , Saver JL . Identifying stroke in the field: prospective validation of the Los Angeles Prehospital Stroke Screen (LAPSS). Stroke 2000;31(1):71–6. 10.1161/01.STR.31.1.71 10625718

[12] Patel MD , Rose KM , O’Brien EC , Rosamond WD . Prehospital notification by emergency medical services reduces delays in stroke evaluation: findings from the North Carolina Stroke Care Collaborative. Stroke 2011;42(8):2263–8. 10.1161/STROKEAHA.110.605857 21659638 PMC3970287

[13] The breakthrough series: IHI’s collaborative model for achieving breakthrough improvement. IHI Innovation Series white paper. Boston (MA): Institute for Healthcare Improvement; 2003. http://www.ihi.org/knowledge/Pages/IHIWhitePapers/TheBreakthroughSeriesIHIsCollaborativeModelforAchievingBreakthroughImprovement.aspx. Accessed March 12, 2013.

[14] Schouten LMT , Hulscher MEJL , Van Everdingen JJE , Huijsman R , Grol RPTM . Evidence for the impact of quality improvement collaboratives: systematic review. BMJ 2008;336(7659):1491–4. 10.1136/bmj.39570.749884.BE 18577559 PMC2440907

[15] O’Neill HJ , Coe LJ , Magdon-Ismail Z , Schwamm LH . Implementing a state-based stroke quality improvement collaborative: the Massachusetts experience. Crit Pathw Cardiol 2012;11(3):114–22. 10.1097/HPC.0b013e31825e12a6 22825531

[16] Acker JE , Pancioli AM , Crocco TJ , Eckstein MK , Jauch EC , Larrabee H , Implementation strategies for emergency medical services within stroke systems of care: a policy statement from the American Heart Association/American Stroke Association Expert Panel on Emergency Medical Services Systems and the Stroke Council. Stroke 2007;38(11):3097–115. 10.1161/STROKEAHA.107.186094 17901393

[17] Sahni R . Acute stroke: implications for prehospital care. National Association of EMS Physicians Standards and Clinical Practice Committee. Prehosp Emerg Care 2000;4(3):270–2. 10.1080/10903120090941317 10895924

[18] Myers JB , Slovis CM , Eckstein M , Goodloe JM , Isaacs SM , Loflin JR , Evidence-based performance measures for emergency medical services systems: a model for expanded EMS benchmarking. Prehosp Emerg Care 2008;12(2):141–51. 10.1080/10903120801903793 18379908

[19] Cone DC . Knowledge translation in the emergency medical services: a research agenda for advancing prehospital care. Acad Emerg Med 2007;14(11):1052–7. 17761547 10.1197/j.aem.2007.06.014

[20] Kleindorfer D , Hill MD , Woo D , Tomsick T , Pancioli A , Kissela B , A description of Canadian and United States physician reimbursement for thrombolytic therapy administration in acute ischemic stroke. Stroke 2005;36(3):682–7. 10.1161/01.STR.0000155742.46437.65 15692114

[21] EMS Stroke Point of Entry Plan (S-PEP). Boston (MA): Massachusetts Department of Public Health, Bureau of Health Care Safety and Quality, Office of Emergency Medical Services; 2005. http://www.mass.gov/eohhs/docs/dph/emergency-services/ambulance-stroke-point-of-entry.pdf. Accessed March 12, 2013.

[22] Langley G , Nolan K , Nolan T , Norman C , Provost L . The improvement guide: a practical approach to enhancing organizational performance. 2nd edition. San Francisco (CA): Jossey-Bass Publishers; 2009.

[23] Behrens S , Daffertshofer M , Interthal C , Ellinger K , VanAckern K , Hennerici M . Improvement in stroke quality management by an educational programme. Cerebrovasc Dis 2002;13(4):262–6. 10.1159/000057853 12011551

[24] Gladstone DJ , Rodan LH , Sahlas DJ , Lee L , Murray BJ , Ween JE , A citywide prehospital protocol increases access to stroke thrombolysis in Toronto. Stroke 2009;40(12):3841–4. 10.1161/STROKEAHA.108.540377 19875743

[25] Wojner-Alexandrov AW , Alexandrov AV , Rodriguez D , Persse D , Grotta JC . Houston paramedic and emergency stroke treatment and outcomes study (HoPSTO). Stroke 2005;36(7):1512–8. 10.1161/01.STR.0000170700.45340.39 15961712

[26] Bray JE , Martin J , Cooper G , Barger B , Bernard S , Bladin C . An interventional study to improve paramedic diagnosis of stroke. Prehosp Emerg Care 2005;9(3):297–302. 10.1080/10903120590962382 16147479

[27] Ramanujam P , Guluma KZ , Castillo EM , Chacon M , Jensen MB , Patel E , Accuracy of stroke recognition by emergency medical dispatchers and paramedics — San Diego experience. Prehosp Emerg Care 2008;12(3):307–13. 10.1080/10903120802099526 18584497

[28] Williams I , Mears G , Raisor C , Wilson J . An emergency medical services toolkit for improving systems of care for stroke in North Carolina. Prev Chronic Dis 2009;6(2):A67. http://www.cdc.gov/pcd/issues/2009/apr/08_0175.htm. 19289010 PMC2687873

[29] Mears GD , Pratt D , Glickman SW , Brice JH , Glickman LT , Cabañas JG , The North Carolina EMS Data System: a comprehensive integrated emergency medical services quality improvement program. Prehosp Emerg Care 2010;14(1):85–94. 10.3109/10903120903349846 19947872

[30] ØVretveit J , Bate P , Cleary P , Cretin S , Gustafson D , McInnes K , Quality collaboratives: lessons from research. Qual Saf Health Care 2002;11(4):345–51. 10.1136/qhc.11.4.345 PMC1757995 12468695

