# Supplementary material for: The Massachusetts Emergency Medical Service Stroke Quality Improvement Collaborative, 2009–2012
Source: Prev Chronic Dis. 2013 Sep 26;10:E161. doi: 10.5888/pcd10.130126 (PMC3786607; doi:10.5888/pcd10.130126)
Supplement: Supplementary file 1 [file 13_0126_01.doc]

**Appendix 1. EMS Stroke Quality Improvement Collaborative Focus Group Question Guide**

1. To begin, can you please tell us your name, and what organization you work for?
2. What were some of the reasons why your organization decided to participate in the EMS Stroke Quality Improvement Initiative?
   1. What are some of the reasons for your continued involvement in the Initiative? What are the benefits?
3. What have been some successes you’ve been able to achieve as a result of your participation in this Initiative?
   1. Please describe how the Initiative has helped to achieve this?
4. What has been your experience and level of satisfaction with the following aspects of the Initiative?
   1. Recruitment period/initial introduction to the project and data collection tool
   2. Initial training of EMTs at your service
   3. Site visits
   4. Conference calls
   5. Time spent on case review (how much time do you typically spend? Too frequent?)
   6. Time spent on Initiative participation (indicate what is included in “participation” for the particular respondent)
   7. Date, location and time of meetings
   8. Topics of meetings
   9. Effectiveness of meetings
   10. Group dynamic
   11. Collaborative nature/meetings
5. What have you found to be the most challenging about participating in this Initiative?
   1. Time commitment
   2. Lack of training/information needed to make changes within my organization
   3. Low stroke volume (is it the best example to drive change/improvements?/relevance to a particular service?)
   4. Scheduling of meetings (absenteeism)
   5. Level of Support/buy-in from bosses/administrators
6. How do you use the data that this initiative provides to you, if at all? (NOTE: If they have not used the data at all, we will want to know this – the May 10^th^ group may have less use)
   1. How use?
   2. How often?
   3. In what settings?
   4. Presented to whom?
7. Has your participation in/work in this Stroke QI Initiative impacted relationships more broadly?
   1. With the receiving Emergency Department(s)/Hospital(s) Staff?
   2. With regional EMS directors/staff?
   3. With the surrounding community?
   4. With OEMS?
   5. With other EMS agencies?
   6. How are these interactions/relationships different now than in the past (before the Initiative)? Please describe.
8. Have you used the concepts and/or data collection from the Collaborative for QI efforts outside of stroke (i.e. for other conditions/acute events)?
   1. How?
   2. What have the concepts/data helped you to do better?
   3. What other areas in your EMS work do you think could be improved by applying these concepts/using the data?
   4. If you have not yet used the concepts/data more broadly, why?
9. What would be essential to continuing your involvement in the Stroke Initiative?
   1. What is the minimum needed?
   2. What could be managed without?
10. What do you see as the next steps in improving this initiative?
11. Is there anything else you’d like to share with us today?
